# Gesture Recognition by Ensemble Extreme Learning Machine Based on Surface Electromyography Signals

**DOI:** 10.3389/fnhum.2022.911204

**Published:** 2022-06-16

**Authors:** Fulai Peng, Cai Chen, Danyang Lv, Ningling Zhang, Xingwei Wang, Xikun Zhang, Zhiyong Wang

**Affiliations:** Medical Rehabilitation Research Center, Shandong Institute of Advanced Technology, Chinese Academy of Sciences, Jinan, China

**Keywords:** sEMG signal, gesture recognition, extreme learning machine, machine learning, feature selection

## Abstract

In the recent years, gesture recognition based on the surface electromyography (sEMG) signals has been extensively studied. However, the accuracy and stability of gesture recognition through traditional machine learning algorithms are still insufficient to some actual application scenarios. To enhance this situation, this paper proposed a method combining feature selection and ensemble extreme learning machine (EELM) to improve the recognition performance based on sEMG signals. First, the input sEMG signals are preprocessed and 16 features are then extracted from each channel. Next, features that mostly contribute to the gesture recognition are selected from the extracted features using the recursive feature elimination (RFE) algorithm. Then, several independent ELM base classifiers are established using the selected features. Finally, the recognition results are determined by integrating the results obtained by ELM base classifiers using the majority voting method. The Ninapro DB5 dataset containing 52 different hand movements captured from 10 able-bodied subjects was used to evaluate the performance of the proposed method. The results showed that the proposed method could perform the best (overall average accuracy 77.9%) compared with decision tree (DT), ELM, and random forest (RF) methods.

## Introduction

Hand gesture recognition provides a natural and convenient human–computer interaction mode for rehabilitation training, virtual games, sign language translation, and many other applications. There are many approaches to recognize hand gestures, such as computer vision, motion capture gloves, surface electromyography (sEMG) signals, and even the combination of sEMG with near-infrared (NIR) (Nsugbe et al., 2020) and electroencephalography (EEG) (Nsugbe et al., 2021). Among the methods, gesture recognition based on sEMG signals has been widely studied in recent the years due to the fact that the process of capturing sEMG signals is not affected by the variations in light, position, and orientation of the hand (Zhang et al., 2019). As one kind of neural electrical activity signal, sEMG signals can be recorded non-invasively and conveniently by sEMG electrodes attached on the skin surface.

The critical part for applications using sEMG signals as intermediate media is to distinguish sEMG signals collected from different gestures accurately. Recently, the pattern recognition technology has become the primary method used to recognize the motion intention, in which, a classifier trained by supervised learning is utilized to map sEMG signals to one of the predefined classes that correspond to different motions (Chen J. et al., 2020). The process of motion intention recognition usually consists of several key procedures, including data acquisition, preprocessing, feature extraction and selection, and classification (Jaramillo-Yánez et al., 2020). Various methods were proposed and tried to enhance the gesture recognition performance by the researchers for the past few years. The existing gesture recognition approaches could be roughly grouped into feature-based and time series-based methods depending on whether the features need to be manually extracted beforehand.

In the feature-based method, features are first extracted manually by experience and then fed into the classification models, which are mainly constructed based on the machine learning algorithms. The diverse features can be roughly divided into four categories: time domain features, frequency domain features, time–frequency domain features, and non-linear features such as fuzzy entropy (FEn) and permutation entropy (PEn) (Mengarelli et al., 2020). Although the suitability of each feature in accurately classifying sEMG signals has been extensively investigated (Du et al., 2010; Phinyomark et al., 2013), there still exists information redundancy among the features inevitably. Thus, feature dimensionality reduction and feature selection techniques are adopted to reduce the feature information redundancy and select the features that mostly contribute to the classification. Riillo et al. (2014) utilized principal component analysis (PCA) to reduce dimensionality of the feature vector after the feature extraction stage. Phinyomark et al. (2012) investigated the properties of a set of 37 time and frequency domain features, showing that considerable levels of recognition accuracy can be achieved using a small amount of time domain features. In the following classification model construction process, machine learning methods such as support vector machine (SVM), linear discriminant analysis (LDA), random forest (RF), naive Bayes (NB), and artificial neural network (ANN) are mostly used recently. For example, Amirabdollahian and Walters (2017) used a linear kernel SVM to recognize four hand gestures on 26 subjects, obtaining an average recognition accuracy of 94.9% with 8 channels of sEMG signal. Too et al. (2018) performed 17 different hand and wrist gesture classification based on the sEMG signals from Ninapro database by SVM classifier. Using the root mean square (RMS) extracted from discrete wavelet transform (DWT) and the average spectrogram energy at each frequency bin as the input feature vector, they achieved the accuracy of 95 and 71.3% for normally-limbed and amputee subjects, respectively. Zhou et al. (2019) used the RF as a classifier to distinguish 12 finger motions based on the nine single features and nine groups of multiple features extracted from Ninapro database sEMG signals. An average motion classification accuracy of 84.11% was obtained and the best result for one subject was 92.94%. Li et al. (2017) applied LDA classifier combining feature smoothing strategy to enhance the motion recognition performance. By analyzing the results of six able-bodied subjects, the accuracy could be improved about 3–5% depending on the different smoothing strategies. Zhang et al. (2019) proposed a real-time hand gesture recognition model based on the ANN and achieved an average recognition rate of 98.7% on 12 subjects with each performing five gestures.

The feature-based methods could achieve quite high recognition accuracy. However, they need to manually extract the features that contain effective motion information from sEMG signals and the extracted features inevitably contain redundant and irrelevant information. To avoid this problem and further improve the gesture recognition accuracy, deep learning methods such as convolutional neural network (CNN), recurrent neural network (RNN), and the modified network architecture were recently utilized by many researchers. The inputs of deep learning methods are usually sEMG time series rather than manually extracted features. Thus, we will call it time series-based method in this paper. The deep learning models are usually composed of many processing layers and could automatically extract the features of input sEMG time series in multiple levels of abstraction. Shen et al. (2019) presented a method by cascading CNN and stacking ensemble learning, in which CNN acts as the primary classifier to automatically extract sEMG features and the stacking ensemble learning acts as the secondary classifier to integrate the output of primary classifiers. Experiments performed on the Ninapro DB5 dataset demonstrated that the method could perform well in gesture recognition. Considering that the sEMG signals have a sequence nature, Hu et al. (2018) introduced an attention-based hybrid CNN and RNN architecture to take full use of the space and time sequence information from multi-channel sEMG signals. Experiments on five sEMG benchmark datasets revealed that their method could outperform all the reported state-of-the-art methods. The deep learning method could improve gesture recognition performance. However, the constructed models usually have a huge number of parameters, which need a lot more training samples to obtain a relatively high classification performance. To reduce the number of model parameters, Chen L. et al. (2020) developed a compact CNN model (named EMGNet) consisting of four convolutional layers and a max-pooling layer. The experiments performed on the Ninapro DB5 dataset and MYO dataset demonstrated the efficacy of their methods. Although the quantity of model parameter could be reduced by some model compression techniques, it is still much more than that of the models built by traditional machine learning methods, which is a challenge both to the training sample and to the computing platform.

To achieve a relatively high gesture recognition accuracy with as few model parameters as possible, we proposed a new method based on extreme learning machine (ELM) algorithm. The ELM is a single hidden layer feedforward neural network and has been recognized as an effective learning method in many fields (Antuvan et al., 2016; Cene and Balbinot, 2020) due to its fast learning speed and high performance. However, due to the weights of the ELM hidden layer being randomly initialized and remaining unchanged during the training process, the classification boundary may not be an optimal one. Samples located near the classification boundary may be misclassified (Cao et al., 2012). Thus, we proposed a method named ensemble extreme learning machine (EELM) to enhance the accuracy and stability of gesture recognition based on sEMG signals with as few parameters as possible. The main idea of EELM is to first establish multiple independent ELM classifiers and then make an ensemble decision based on the majority voting method. The main procedures are as follows: The input sEMG signals are first preprocessed, and 16 time domain and frequency domain features are extracted from each channel. Second, the features that mostly contribute to gesture recognition are picked out using the recursive feature elimination (RFE) algorithm. Then, several independent ELM classifiers are established by the features selected and their corresponding gesture labels. Finally, the recognition results are determined based on the results obtained by ELM base classifiers using majority voting method.

## Materials and Methods

**Figure 1:**
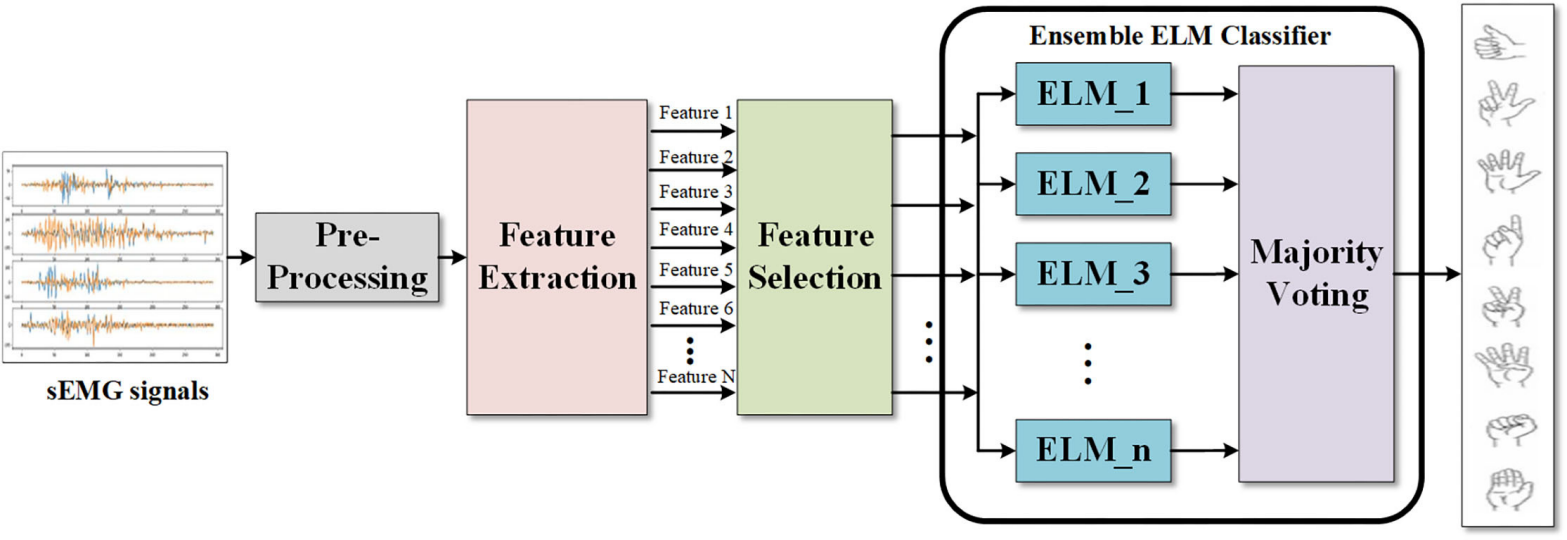
Flowchart of the proposed gesture recognition method.

The flowchart of the proposed gesture recognition method is shown in Figure 1, which consists of several key parts, including signal preprocessing, feature extraction and selection, and ensemble ELM classifier. In the preprocessing section, the multi-channel sEMG signals are first filtered and then processed by sliding windows. The feature extraction and selection process first extract multiple time and frequency domain features, and then, the features that mostly contribute to the result are picked out. The ensemble ELM classifier module consists of multiple ELM base learners and an ensemble learning machine which we employ the majority voting method in this paper.

### Signal Preprocessing

**Figure 2:**
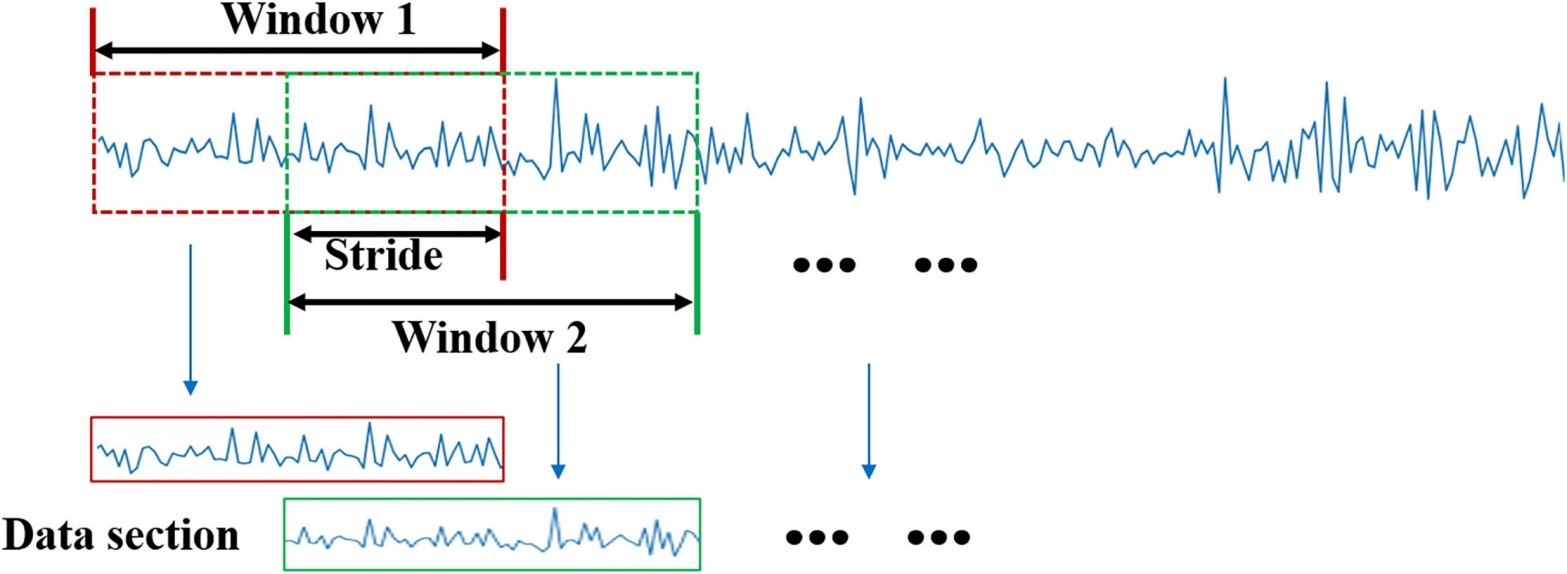
Signal slide window processing.

The sEMG signals are first filtered by Butterworth high pass filter with a 0.5 Hz cutoff frequency to remove the signal DC component. Then, the sliding windows are applied on the processed signals to divide the motion data series into multiple data fragments with fixed lengths, as shown in Figure 2. The window length that is crucial for the real-time system should not be longer than 300 ms according to the previous studies (Shen et al., 2019). In this paper, we set the window length to 300 ms with a stride length of 150 ms. By this window processing, the sequential data are converted into frame data, so that they can be mapped to the action labels.

### Feature Extraction and Selection

The feature extraction procedure is applied to the data sections obtained by window processing. In this paper, 16 time domain and frequency domain features (Toledo-Pérez et al., 2019; Peng et al., 2021) are extracted from each signal channel. The features include mean absolute value (MAV) and its two modified derivatives (MMAV1 and MMAV2), mean absolute value slope (MAVSLP), root mean square (RMS), variance (VAR), waveform length (WL), slope sign change (SSC), zero crossing (ZC), integrated sEMG (IEMG), simple square integral (SSI), median frequency (MDF), peak frequency (PKF), mean frequency (MNF), mean power (MNP), and spectral moment (SM). A description of the features is given in Appendix.

**Figure 3:**
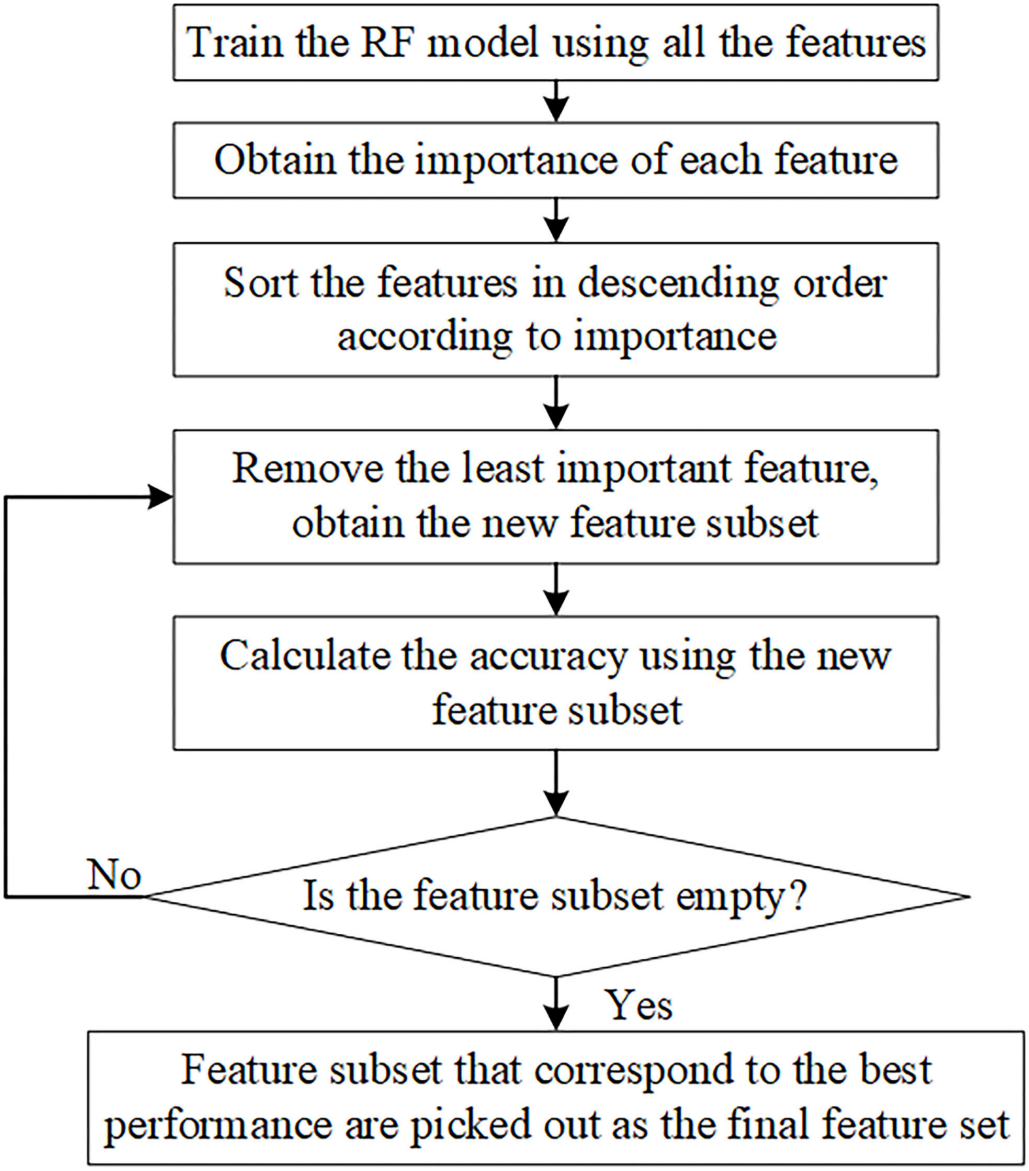
RF-RFE algorithm procedures.

Although the features obtained above could represent the sEMG signals from different dimensions, they are extracted by experience subjectively, which inevitably leads to information redundancy. This will reduce the efficiency of the classification model and increase the computation amount. To alleviate this problem, we use the RF algorithm-based RFE algorithm (Chen et al., 2018), named RF-RFE to select the features that mostly contribute to the recognition results. The RF-RFE is one kind of sequential backward selection method, whose procedures are as follows: First, the model based on RF is trained using the whole training features, obtaining the importance of each feature according to their classification contribution; Second, the features are descending sorted according to the importance; Third, the least important feature is eliminated, and RF classification model is re-trained using the new feature set. The third process was repeated until the feature set is empty. After the RF-RFE, we obtain a list of performance measurement values corresponding to each subset. Finally, the features that correspond to the best performance are picked out as the final feature set. The main procedures of RF-RFE algorithm are shown in Figure 3.

### Ensemble Extreme Learning Machine

#### Extreme Learning Machine

**Figure 4:**
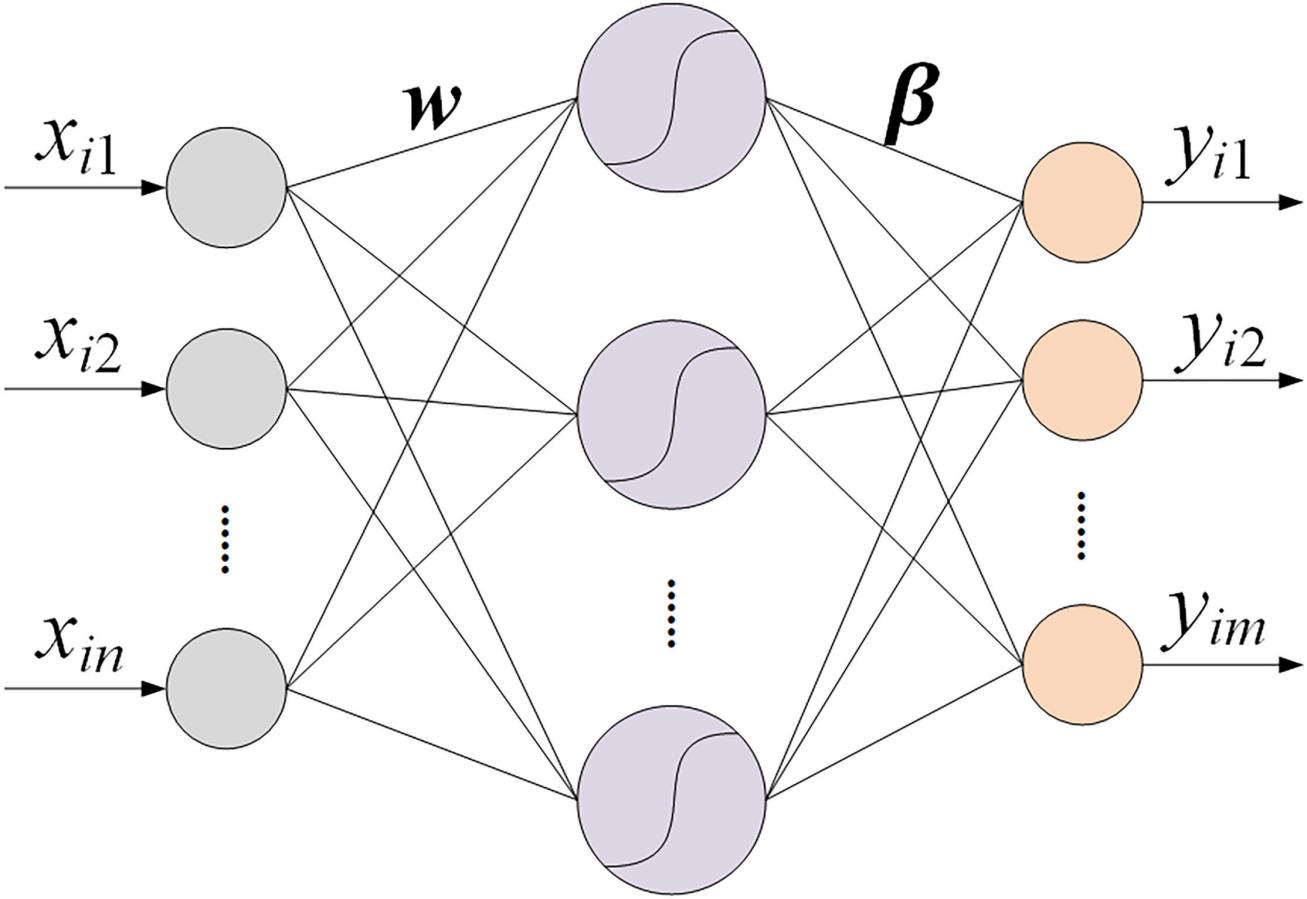
The structure of ELM.

Extreme learning machine (Huang et al., 2006) is one kind of single hidden layer feedforward neural network, which is composed of three layers, named input layer, hidden layer, and output layer. The weights between the input layer and hidden layer are randomly assigned and remain unchanged during the training process. The ELM output weights are determined by an analytical solution. Thus, there is no iteration in the training process, and the training speed of ELM is faster than traditional neural networks. The structure of ELM is shown in Figure 4.

Suppose that $x_{i}={\left[x_{i1},x_{i2},\ldots ,x_{in}\right]}^{T}$, $y_{i}={\left[y_{i1},y_{i2},\ldots ,y_{im}\right]}^{T},\;\left(i=1,2,\ldots ,N\right)$ are the input and expected output, respectively, *g*(*x*) is the activation function. The output of the ELM is as follows:


(1) $$\begin{array}{r} y_{i}=\overset{K}{\underset{j=1}{\sum }}\beta_{j}g\left(w_{j}x_{i}+b_{j}\right),\;i=1,2,\ldots ,N \end{array}$$

where $w_{j}={\left[w_{j1},w_{j2},\ldots ,w_{jn}\right]}^{T}$ is the weight vector between input neurons and the *j*th neuron of hidden layer, $\beta_{j}={\left[\beta_{j1},\beta_{j2},\ldots ,\beta_{jm}\right]}^{T}$ is the weight vector between the *j*th neuron of hidden layer and output neurons. N is the total number of samples. *n, m*, and K are the total number of input, output, and hidden layer neurons, respectively.

The equation above can be rewritten as follows:


(2) $$\begin{array}{r} H\beta =Y \end{array}$$

(3) $$\begin{array}{c} H={\left[\begin{array}{ccc} g(w_{1}x_{1}+b_{1}) & \ldots & g(w_{K}x_{1}+b_{K})\\ \vdots & \ldots & \vdots \\ g(w_{1}x_{N}+b_{1}) & \ldots & g(w_{K}x_{N}+b_{K}) \end{array}\right]}_{N\times K} \end{array}$$

(4) $$\begin{array}{c} \beta ={\left[\begin{array}{c} \beta_{1}^{T}\\ \vdots \\ \beta_{K}^{T} \end{array}\right]}_{K\times m},\;Y={\left[\begin{array}{c} y_{1}^{T}\\ \vdots \\ y_{N}^{T} \end{array}\right]}_{N\times m} \end{array}$$

where *H* is the output matrix of hidden layer. The weight vector *w*_*j*_ and bias *b*_*j*_ are randomly assigned and remain unchanged during training process. The weight vector β is determined by the equation:


(5) $$\begin{array}{r} \beta =H^{+}Y \end{array}$$

where *H*^+^is the Moore–Penrose generalized inverse matrix of matrix *H*.

The procedures of ELM could be summarized following the three steps:

(1) First, determine the number of hidden layer neurons and initialize the weight vector *w*_*j*_ between input and hidden layer and bias *b*_*j*_ randomly;
(2) Second, choose an activation function and calculate the hidden layer output matrix *H*;
(3) Finally, calculate the output weight vector by β = *H*^+^*Y*.

#### Voting-Based Extreme Learning Machine

The ELM algorithm could perform well in the classification of a relatively short training runtime. However, due to that the hidden layer parameters are randomly assigned and remained unchanged, the classification boundary may not be optimal, which will result in misclassification of the samples located near the classification boundary (Cao et al., 2012).

To tackle the issue mentioned above and improve the recognition performance of ELM, we classify the sEMG signals by an ensemble ELM algorithm which first establishes multiple independent ELM classification models and then makes a decision by the majority voting method.

**Figure 5:**
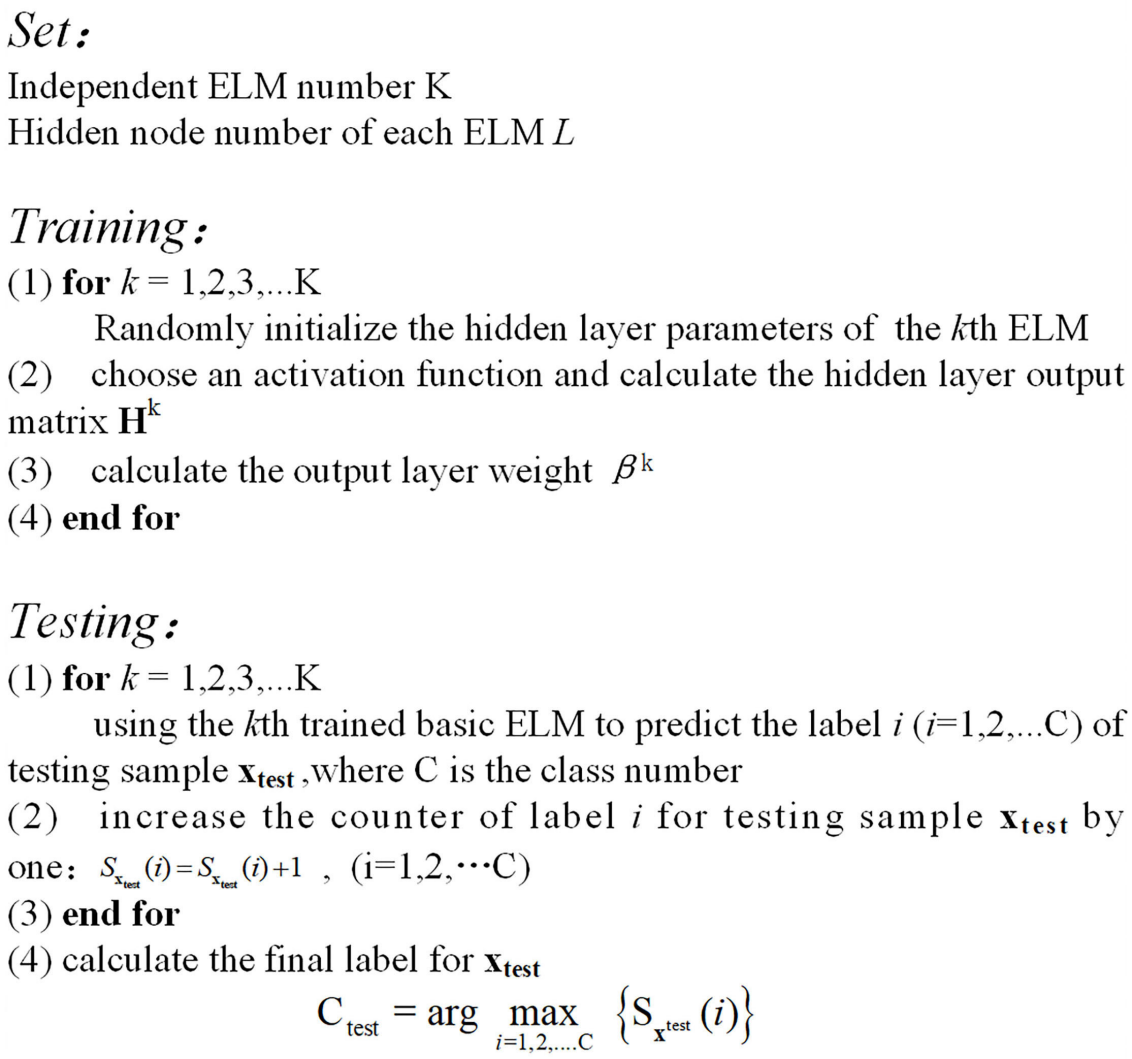
Algorithm procedure of EELM.

The key point of EELM algorithm is to ensure the diversity of ELM base learners. Actually, due to the fact that the hidden parameters of base ELMs are randomly initialized independently, the base ELM learners are different from each other. To make full use of the information from the limited sEMG signals training data, we trained the base ELM learners with all the training samples. In addition, the number of hidden layer neurons is set the same for easy implementation. The procedures of the EELM algorithm based on the majority voting method are shown in Figure 5.

## Experiments and Results

### sEMG Signals Dataset

To evaluate the performance of our proposed method, we used an open-source sEMG signals dataset, named Ninapro DB5 (Pizzolato et al., 2017), which has been widely used in motion recognition research. The Ninapro DB5 dataset contains sEMG signals captured from 10 able-bodied subjects performing 52 different hand movements with each was repeated 6 times. All the movements are divided into three groups (Exercise-A, Exercise-B, and Exercise-C): Exercise-A contains 12 finger basic movements, Exercise-B contains 17 isometric, isotonic hand configurations and basic wrist movements, Exercise-C contains 23 different grasping and functional movements. The sEMG signals in the DB5 database were recorded by double wearable MYO bracelets from the same arm at a 200 Hz acquisition sample rate with a built-in ADC resolution of 8 bit. Each of the MYO could record 8 channels of sEMG signals; therefore, each recording consists of 16-channel time series.

## Results

We first evaluated the efficacy of the feature selection strategy by comparing the classification accuracy before and after the feature selection process. All the extracted 256 features were first used to construct the classification model, and then, the features that mostly contribute to the classification result were selected out by the RF-RFE algorithm. The sEMG signals generated by 52 different movements from subject 5 were used to evaluate the performance of feature selection, in which 70% samples of each movement were used to train the classification model, and the remaining 30% samples were used to test the performance of the constructed models.

**Figure 6:**
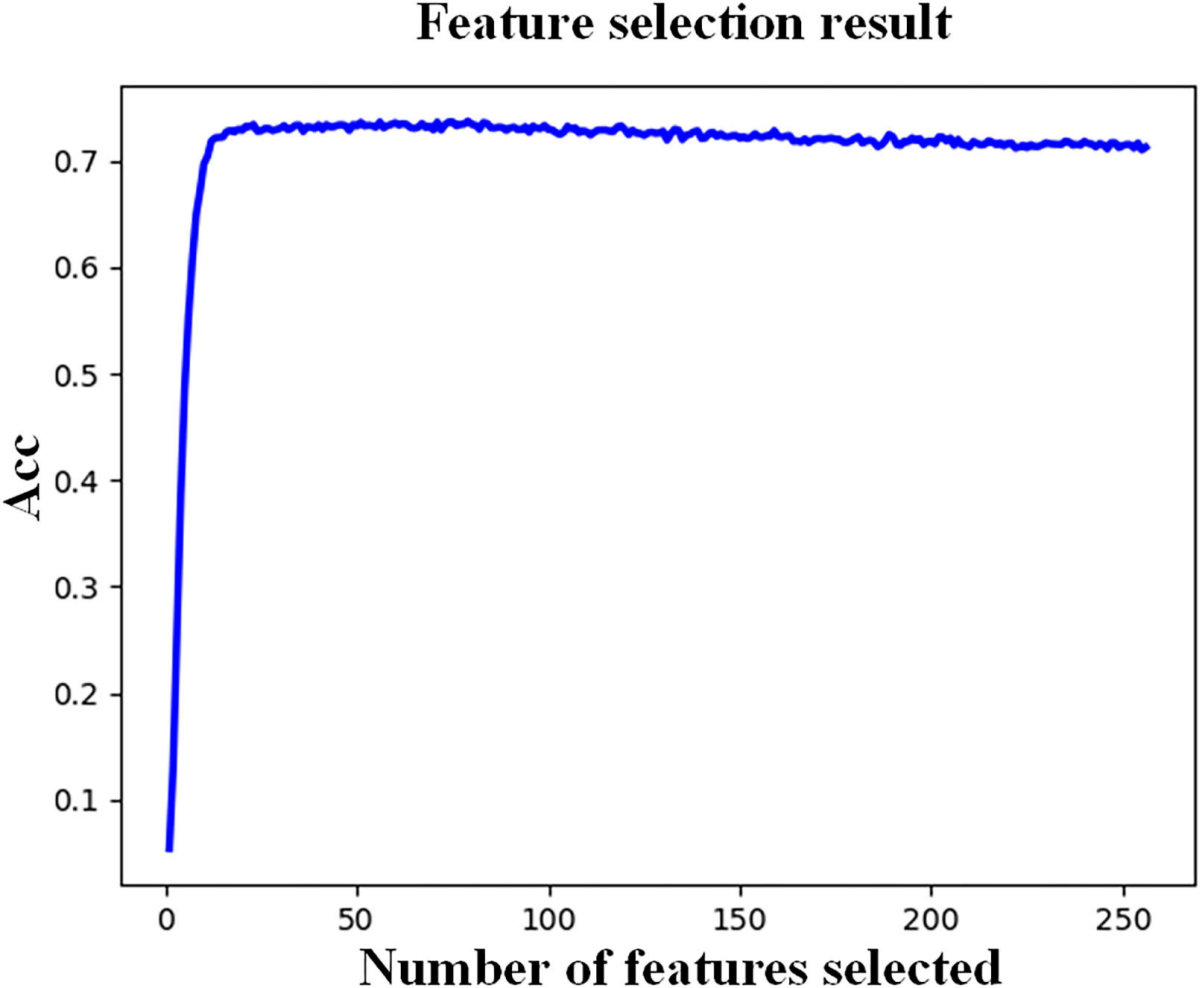
Feature selection result of subject 5.

The feature selection result is shown in Figure 6, in which the x-axis represents the number of features and y-axis represents the classification accuracy based on RF. The classification accuracy reaches the highest when the number of features increases to 79. From Figure 6, we can see that the classification accuracy increases rapidly and then remains nearly unchanged despite the increase in the feature count. To determine the optimal number of selected features, we thoroughly investigate the number of features from 30 to 80 with a step of 10. Finally, we determined the number of features to 40 taking both the accuracy and computational burden into account.

**Figure 7:**
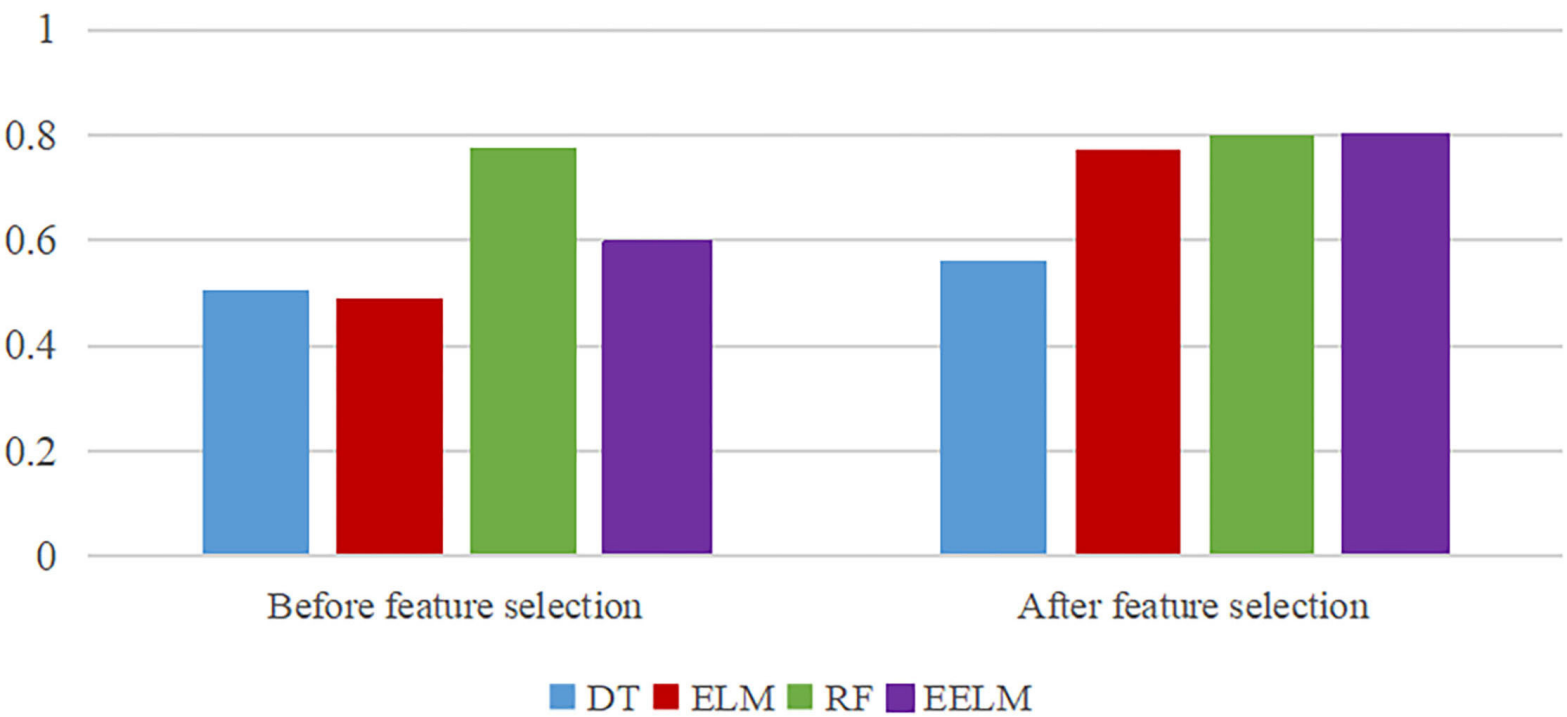
Efficacy of feature selection strategy.

Figure 7 shows the efficacy of feature selection strategy. A total of four classification algorithms including decision tree (DT), ELM, RF, and EELM were used to evaluate the performance. From the figure, the recognition accuracy of ELM and EELM algorithms improved obviously after the feature selection process.

We used DT, ELM, and RF algorithms as the comparison methods to evaluate the recognition performance of the proposed method. sEMG signals in different movement subsets (Exercise-A, Exercise-B, Exercise-C, and Exercise-All) were used to test the performance. The performance indexes including accuracy, F1-score, recall and precision were used to mirror the performance of the methods in motion recognition. About 70% of each movement samples were used to train the classification model, and the remaining 30% were used to test the performance. To ensure the credibility of experiment results, 100 trials were performed for each subject.

Table 1 presents the overall average motion recognition accuracy of all the 10 subjects. The proposed method could achieve the best performance with 87.8, 81.8, and 78.0% accuracy for Exercise-A, Exercise-B, and Exercise-C, respectively. In recognizing the 52 movements considered all together from Ninapro DB5 dataset, the proposed method also performs the best with about 77.9% of recognition accuracy.

**Table 1:** Overall motion recognition accuracy by different methods.

**Methods**	**Exercise-A**	**Exercise-B**	**Exercise-C**	**Exercise-all**
DT	72.8%	61.8%	55.3%	53.6%
ELM	85.3%	79.5%	75.0%	76.0%
RF	85.6%	79.1%	75.8%	77.2%
EELM	87.8%	81.8%	78.0%	77.9%

The statistical analysis was executed to validate the statistical difference among the four methods (DT, ELM, RF, and EELM). We first used the one-way analysis of variance (ANOVA) test to evaluate whether there is a statistical difference among the four methods. Then, a Tukey's honestly significant difference (HSD) test was executed to compare the differences between our proposed method (EELM) and the other three methods. The null hypothesis was rejected (*p* < 0.05) for the ANOVA test, and the Tukey's HSD test (*p* = 0.001 < 0.05) revealed that the proposed method is statistically the best classifier.

**Figure 8:**
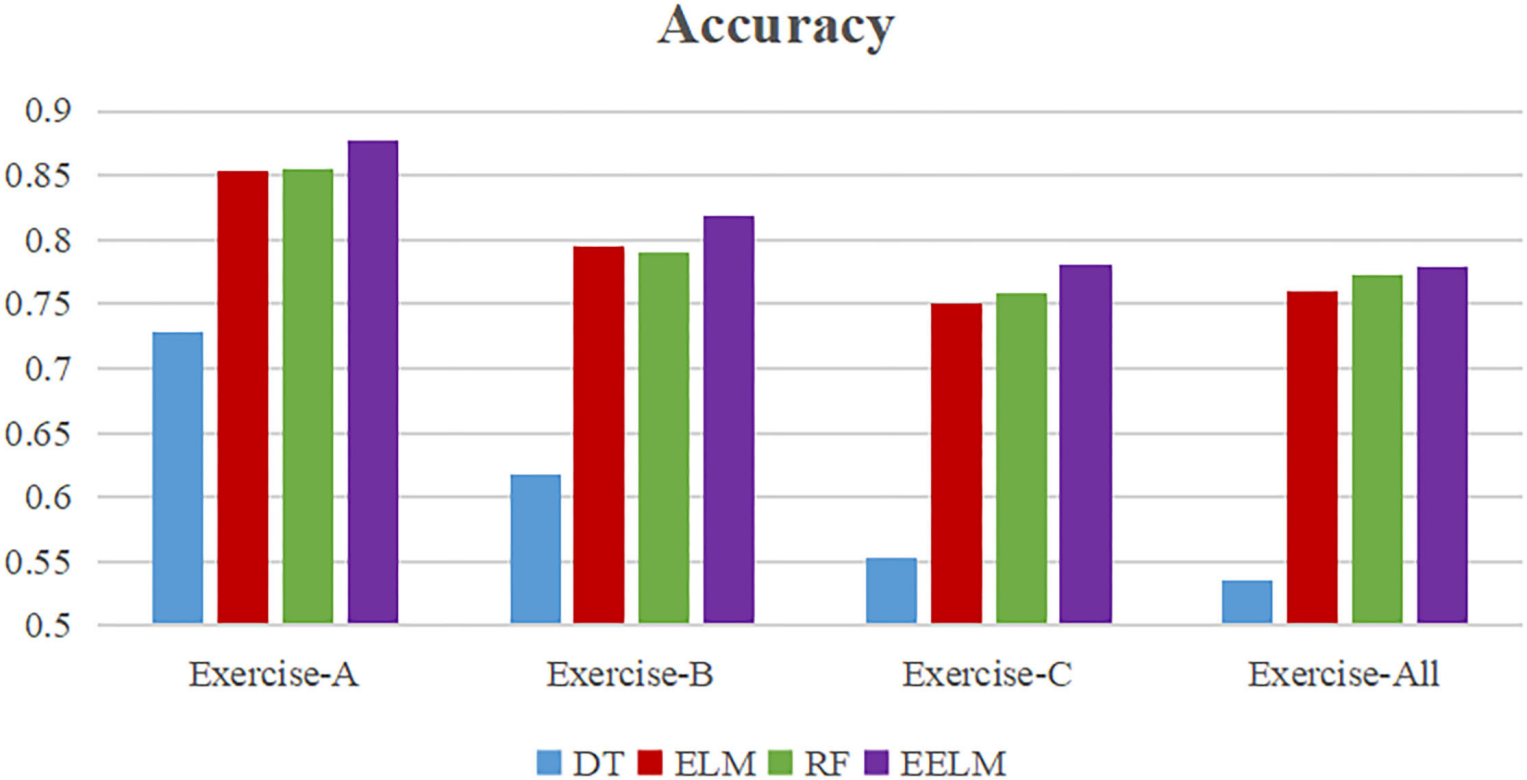
Overall average classification accuracy comparison.

**Figure 9:**
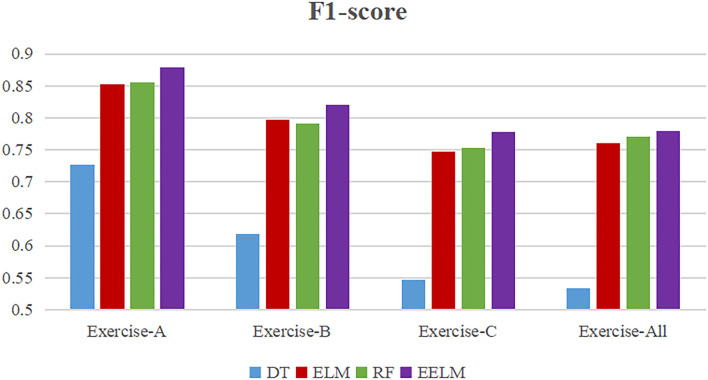
Overall average classification F1-score comparison.

**Figure 10:**
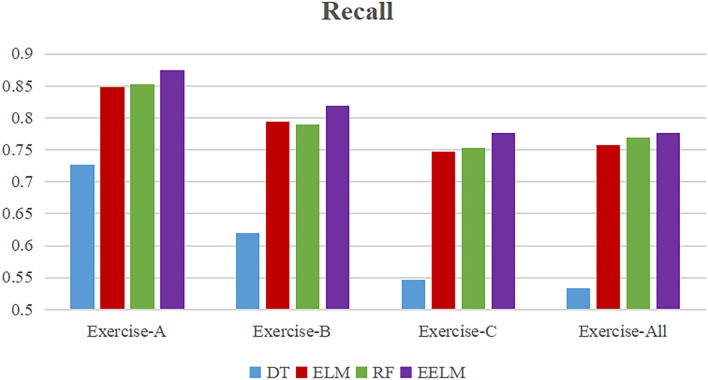
Overall average classification recall comparison.

**Figure 11:**
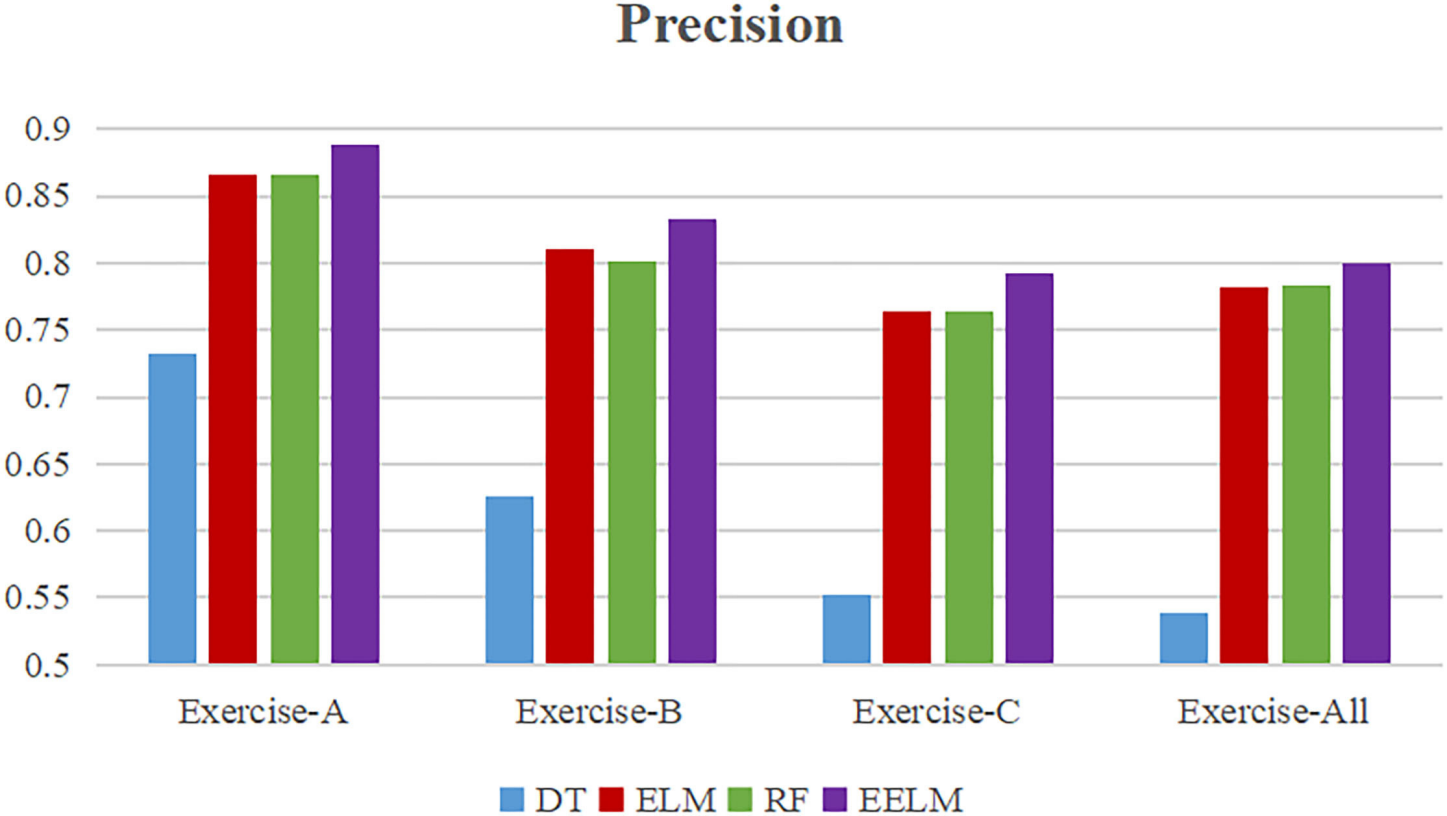
Overall average classification precision comparison.

To present more intuitively, Figures 8–11 show the overall average accuracy, F1-score, recall, and precision, respectively, of all the 10 subjects with different sEMG data subsets. From the figures, we could see that the proposed EELM algorithm performs the best in all the data subsets compared with the other three methods.

**Figure 12:**
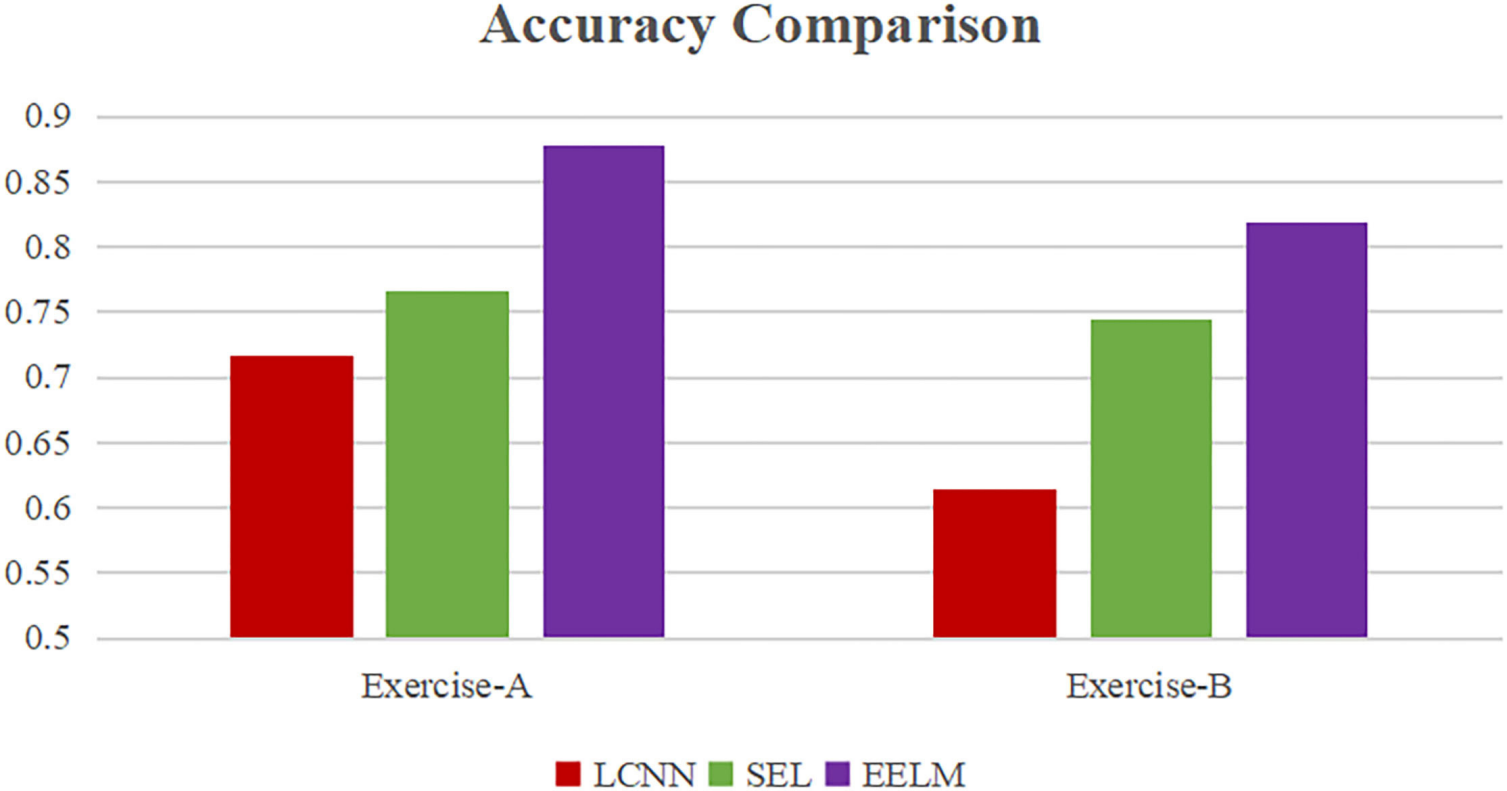
Performance comparison with other existing methods.

To further demonstrate the efficacy of the proposed method, we compared our method with two other existing methods: long short-term memory-CNN (LCNN) presented by Wu et al. (2018) and stacking ensemble learning (SEL) designed by Shen et al. (2019). Both studies used Exercise-A and Exercise-B movement subsets to test the performance of their methods. The performance comparison between our method and theirs is shown in Figure 12. The average accuracy of the LCNN method was 71.7 and 61.4% for Exercise-A dataset and Exercise-B dataset, respectively. The performance of SEL method was better than LCNN, which improved the accuracy by 5 and 13% over LCNN on Exercise-A and Exercise-B, respectively. Our proposed method (EELM) could achieve an average accuracy of 87.8 and 81.8% for Exercise-A and Exercise-B, respectively, performing better than the other two existing methods.

**Figure 13:**
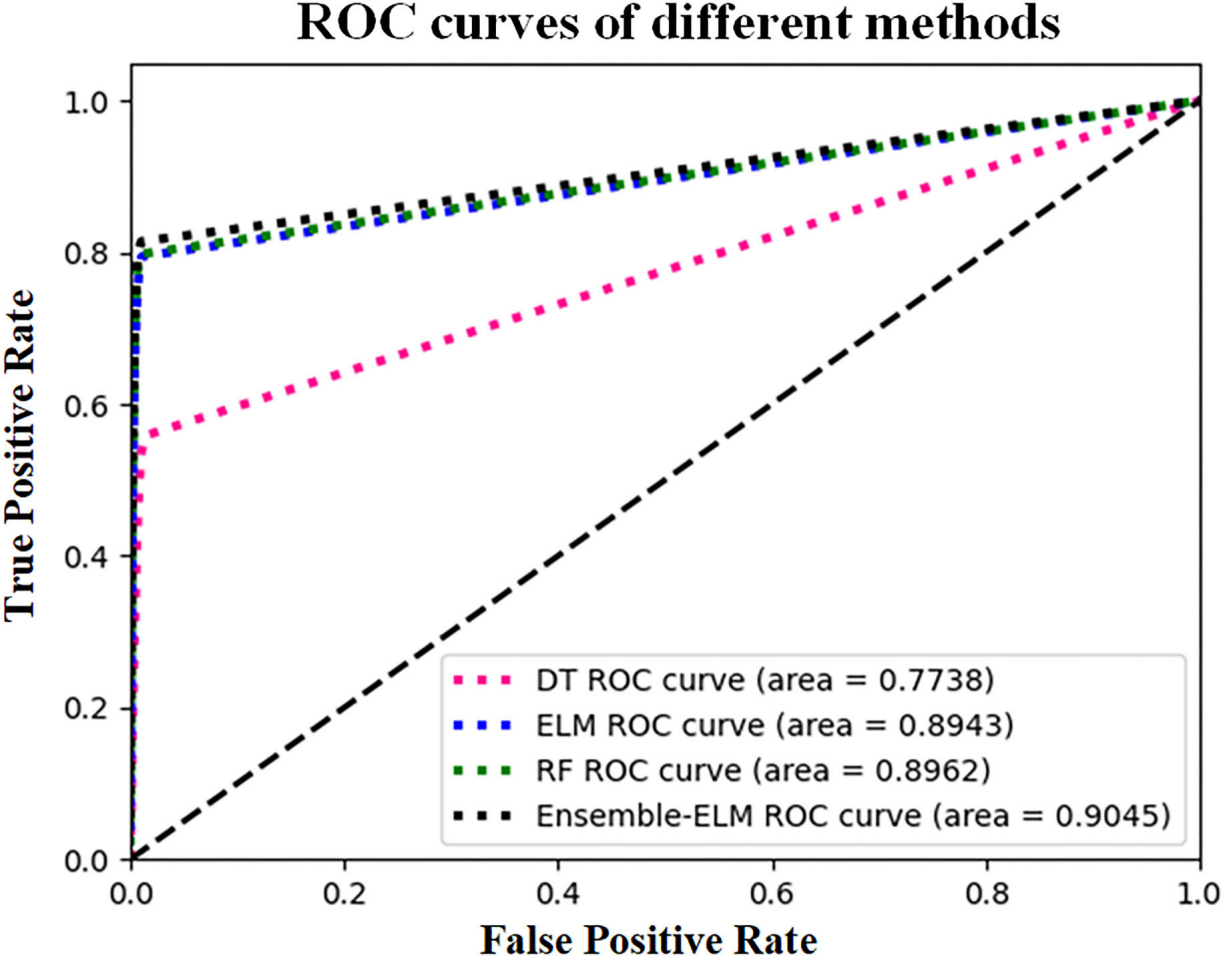
ROC curves and AUC values of different classification methods.

The receiver operating characteristic (ROC) and the area under the curve (AUC) were also used to verify the performance of the proposed method. Figure 13 shows the ROC curves and AUC values obtained by different methods, where the larger AUC value represents the better classification results.

To evaluate the real-time applicability, we further investigated the run time of the EELM method in recognizing gestures. The run time of the constructed model was tested on the datasets from subject 1 in Exercise-A, Exercise-B, and Exercise-C. The test sample size of Exercise-A, Exercise-B, and Exercise-C was *N* = 464, *N* = 717, and *N* = 1,014, respectively. A total of 100 trials were performed to enhance the result credibility. The experiments were run on a laptop equipped with Intel Core i7-8565U CPU @1.80GHz and 8GB RAM. The run time of the other three methods is also presented in Table 2 for comparison. From Table 2, we could find that our proposed EELM is faster than RF.

**Table 2:** Run time of each method in recognizing gestures (ms).

**Methods**	**Exercise-A**	**Exercise-B**	**Exercise-C**
	**(*N* = 464)**	**(*N* = 717)**	**(*N* = 1,014)**
DT	0.6	0.7	0.8
ELM	10.8	13.0	24.0
RF	116.6	133.4	428.1
EELM	81.2	108.3	205.6

## Discussion

Gesture recognition based on sEMG signals has been widely studied in the recent years. Depending on whether the feature information needs to be manually extracted beforehand, the existing gesture recognition approaches could be roughly grouped into feature-based and time series-based methods. In the feature-based method, features that represent motion information are first extracted manually based on experience and then fed into the classification models which are constructed based on the machine learning algorithm. The information in the extracted features is usually redundant, and thus, feature selection techniques are adopted to alleviate this problem by many studies. While in the time series-based method, the sEMG time series are directly inputted to the recognition models without manual feature extraction. The classification models of the time series-based method are usually constructed based on deep learning algorithm. Although the deep learning algorithm could achieve a relatively high recognition performance, its computational burden is heavy due to its huge number of parameters.

The ELM which is a single hidden layer feedforward neural network has been recognized as an effective learning method in many fields with fast learning speed and high performance. However, the accuracy and stability cannot fulfill some actual applications such as prosthetic hand control. Since the ELM hidden parameters are randomly assigned, the classification boundary is not optimal, and samples located near the boundary may be misclassified. In this paper, we integrated the results of several ELM base learners by the majority voting method to enhance the motion recognition accuracy and stability. The prerequisite for integrating the ELM base learners is to guarantee the diversity of each base learner. Actually, the randomly initialized hidden layer parameters meet the above-mentioned requirements that the base ELM learners are different from each other. Experiments proved that the EELM method outperforms traditional ELM in recognizing gestures based on the sEMG signals.

In the feature extraction and selection process, 256 features were extracted from each subject with 16 features from each sEMG channel. The extracted features were then simplified by the RF-RFE feature selection algorithm, reserving the features that mostly contribute to the recognition results. In Figure 6, classification accuracy increases rapidly first and then remains nearly unchanged despite the increase in the selected feature count. Although the performance reaches the best using 79 features, we determined the number of finally selected features to 40, taking both the accuracy and computational burden into account by full insight on the number of features from 30 to 80 with a step of 10. When coupled with the Figure 7 information, it leads to some conclusions that the feature selection strategy could enhance the gesture recognition performance across all the four classification methods (DT, ELM, RF, and EELM). The EELM could achieve the highest recognition accuracy after the feature selection process. However, the RF performs better than EELM before feature the selection process. It may be because that the RF model construction process essentially contains the feature selection procedure, the most important features are always in the first place to be selected out and used, reducing the effect of other least important features, whereas, in the EELM model construction process, all the features including the irrelevant and redundant features contribute equally to the EELM model construction, degrading the performance of EELM model. By the feature selection process, the least important features could be rejected, and thus, the performance of EELM could be improved. From Figure 7, it can be seen that the performance of DT and RF improved less by the feature selection strategy. In contrast, the ELM and EELM significantly improved after the feature selection process, which is consistent with our explanation.

To further evaluate the performance of the proposed method, sEMG signals in different movement subsets (Exercise-A, Exercise-B, Exercise-C, and Exercise-All) were used to test the performance. Table 1 and Figures 8–11 show the overall performances of the four methods in movement classification using different evaluation parameters. From the presented results, EELM combining feature selection process performs the best in all the data subsets, and the statistical analysis verified this. We also compared our method with the other two existing methods: LCNN and SEL methods. The performance comparison between our method and theirs is shown in Figure 12, demonstrating that our method performs better than the other two existing methods. The real-time applicability of the proposed method was also investigated by testing the algorithm run time in recognizing gestures. The experimental results are presented in Table 2, demonstrating that the EELM is faster than RF, inferring that the real-time capability is acceptable for real-time application scenarios.

## Conclusion

In summary, this paper proposed a method combining feature selection and EELM algorithms to improve the motion recognition performance based on sEMG signals. First, the input sEMG signals are preprocessed by a sliding window and 16 features in each channel are extracted. Second, the features that mostly contribute to gesture recognition are picked out using the RF-RFE algorithm. Third, several independent ELM classifiers are established by the features selected. Finally, the recognition results are determined by integrating the results obtained by ELM base classifiers using the majority voting method. Ninapro DB5 dataset with 52 different movements was used to evaluate the performance of the proposed method. The results showed that the motion recognition performance could be improved evidently by the RF-RFE feature selection process. In addition, the proposed method could achieve the best accuracy (77.9%) compared with DT, ELM, and RF methods. The research achievement proved that our proposed method could effectively enhance the gesture recognition performance based on sEMG signals. According to the findings of this paper, our further work will focus on improving the recognition accuracy in distinguishing similar gestures and exploring a universal gesture recognition model across different subjects to easily migrate gesture recognition system between subjects quickly.

## Data Availability Statement

Publicly available datasets were analyzed in this study. This data can be found at: http://ninapro.hevs.ch/DB5_DoubleMyo.

## Author Contributions

FP: guarantor of integrity of entire study, study design, and manuscript preparation. CC and XZ: study concept. XW: literature research. NZ and ZW: data analysis. DL: manuscript editing and manuscript revision and review. All authors have participated in this study and consent to publish this article in journal.

## Funding

The research was supported by the project of Natural Science Foundation of Shandong Province (ZR2020QF024, ZR2021QH290), Jinan 20 Universities (2019GXRC040), Jinan 5150 Program for Talents Introduction, Major Basic Research Project of Shandong Natural Science Foundation (ZR2021ZD40), Key Research and Development Plan of Shandong Province (2021CXGC011304), and Shandong Institute of Advanced Technology, Chinese Academy of Sciences (YJZX003, YQCX20220106).

## Conflict of Interest

The authors declare that the research was conducted in the absence of any commercial or financial relationships that could be construed as a potential conflict of interest.

## Publisher's Note

All claims expressed in this article are solely those of the authors and do not necessarily represent those of their affiliated organizations, or those of the publisher, the editors and the reviewers. Any product that may be evaluated in this article, or claim that may be made by its manufacturer, is not guaranteed or endorsed by the publisher.
