# Supplementary material for: Gesture Recognition by Ensemble Extreme Learning Machine Based on Surface Electromyography Signals
Source: Front Hum Neurosci. 2022 Jun 16;16:911204. doi: 10.3389/fnhum.2022.911204 (PMC9243223; doi:10.3389/fnhum.2022.911204)
Supplement: Supplementary file 1 [file Data_Sheet_1.pdf]

Appendix

(1) Means Absolute Value(MAV)

MAV is one of the most used time domain statistics feature, which is the mean absolute values of signal amplitude within the sliding window. For a segments of N samples, it is described as

$$\mathrm{MAV} = \frac{1}{N}\sum_{k=1}^{N}|s(k)|$$

where *s*(*k*) is the *k*-th sample.

(2) Modified Means Absolute Value type1(MMAV1)

MMAV1 is a generalization of MAV, where the *s*(*k*) in MAV is weighted by the function w_k.

$$\mathrm{MMAV1} = \frac{1}{N}\sum_{k=1}^{N} w_k|s(k)|$$

where w_k is defined as

$$w_k = \begin{cases} 1, & 0.25N \le k \le 0.75N \\ 0.5, & otherwise \end{cases}$$

(3) Modified Means Absolute Value type2(MMAV2)

MMAV2 is another generalization of MAV, where the *s*(*k*) in MAV is weighted by a continuous function w_k, improving the smoothness of the weighted function.

$$\mathrm{MMAV2} = \frac{1}{N}\sum_{k=1}^{N} w_k|s(k)|$$

where w_k is

$$w_k = \begin{cases} 1, & 0.25N \le k \le 0.75N \\ \frac{4k}{N}, & k < 0.25N \\ 4(k-N)/N, & otherwise \end{cases}$$

(4) Mean Absolute Value Slope (MAVSLP)

MAVSLP is a modified version of MAV, which is calculated by the differences between the MAVs of adjacent segments.

$$\mathrm{MAVS} = MAV_{k+1} - MAV_k$$

(5) Root Mean Square (RMS)

RMS is modeled as the amplitude modulated Gaussian random process, which is related to the constant force and contraction without fatigue. It is defined as

$$\mathrm{RMS} = \sqrt{\frac{1}{N}\sum_{k=1}^{N} s(k)^2}$$

(6) Variance of sEMG (VAR)

VAR is the power of the sEMG signal, which is the mean value of the square of the deviation of the variable.

$$\mathrm{VAR} = \frac{1}{N-1}\sum_{k=1}^{N} s(k)^2$$

(7) Waveform Length (WL)

WL presents the complexity of the waveform which is the cumulative length of the signal waveform.

$$\mathrm{WL} = \sum_{k=2}^{N} |s(k) - s(k-1)|$$

(8) Slope Sign Change (SSC)

SSC is a method that represents the frequency information of signal, it is the number of times that a slope sign change occurs.

$$\mathrm{SSC} = \sum_{k=2}^{N} [f[(s(k) - s(k-1)) * (s(k) - s(k+1))]]$$

Where $f(x)$is

$$f(x) = \begin{cases} 1, & if\ x \geq threshold \\ 0, & otherwise \end{cases}$$

(9) Zero Crossing (ZC)

ZC is the number of times that the amplitude value of sEMG signal crosses zero.

$$\mathrm{ZC} = \sum_{k=1}^{N-1} [sgn(x(k) \times (k+1)) \cap |x(k) - x(k+1)| \geq T]$$

(10) Integrated sEMG (IEMG)

IEMG is defined as a summation of the absolute values of signal amplitude.

$$\mathrm{IEMG} = \sum_{k=1}^{N} |(s(k)|$$

(11) Simple Square Integral (SSI)

SSI represents the energy of the sEMG signal, which is defined as

$$\mathrm{SSI} = \sum_{k=1}^{N} |(s(k)|^2$$

(12) Median Frequency (MDF)

MDF is a frequency at which the power spectrum can be divided into two equal regions.

$$\sum_{i=1}^{MDF} P_i = \sum_{i=MDF}^{N} P_i = \frac{1}{2}\sum_{i=1}^{N} P_i$$

where P_i is the power spectrum of frequency bin *i* and N is the length of the frequency bin *i*.

(13) Peak Frequency (PKF)

PKF is the frequency at which the power spectrum is maximum.

$$\mathrm{PKF} = \max\,(P_i)$$

(14) Mean Frequency (MNF)

MNF is the quotient obtained by dividing the sum of the signal power spectrum multiplied with frequency by total sum of the power spectrum.

$$\mathrm{MNF} = \sum_{i}^{N} P_i\, f_i \bigg/ \sum_{i}^{N} P_i$$

where P_i and f_i are the power and frequency of the spectrum at bin *i*.

(15) Mean Power (MNP)

MNP is the mean EMG power.

$$\mathrm{MNP} = \left(\sum_{i}^{N} P_i\right)/N$$

(16) Spectral Moment (SM)

SM is another EMG power spectrum statistical analysis way.

$$\mathrm{SM} = \sum_{i}^{N} P_i\, f_i$$
